# Adolescent male and female rats show enhanced latent inhibition of conditioned fear compared to adult rats

**DOI:** 10.3389/fnbeh.2025.1636674

**Published:** 2025-10-22

**Authors:** Christina J. Perry, Ricky John, Han B. Trinh, Brandon K. Richards, Katherine D. Drummond, Chun Hui J. Park, Jee Hyun Kim

**Affiliations:** ^1^School of Psychological Sciences, Centre for Emotional Health, Macquarie University, North Ryde, NSW, Australia; ^2^Florey Institute of Neuroscience and Mental Health, Parkville, VIC, Australia; ^3^Florey Department of Neuroscience and Mental Health, The University of Melbourne, Parkville, VIC, Australia; ^4^School of Medicine, IMPACT-The Institute for Mental and Physical Health and Clinical Translation, Deakin University, Geelong, VIC, Australia

**Keywords:** adolescence, pubertal, estrous, memory, psychology, resilience, anxiety disorders, development

## Abstract

**Introduction:**

Latent inhibition is diminished associative memory because of pre-exposure to the conditioned stimulus without any consequences. Latent inhibition likely plays a significant role in the ontogeny of anxiety disorders, contributing to why anxiety disorders are particularly prevalent in adolescence. Therefore, the present study examined latent inhibition of conditioned fear in adolescent and adult rats of each sex. Given that adolescence is associated with deficits in fear extinction, we hypothesized that latent inhibition will be impaired in adolescents compared to adults and expected females to show age-specific estrous cycle effects.

**Methods:**

On day 1, male (Experiment 1) and female (Experiment 2) rats were placed in fear conditioning chambers. Half of the rats received pre-exposure to the tone cue while the other half received nothing. On day 2, all rats were placed back in the same chambers and exposed to three cue-footshock pairings. Latent inhibition was tested on day 3 with 20 presentations of the cue by itself in the same chamber.

**Results:**

We unexpectedly observed enhanced latent inhibition in adolescents compared to adults in both male and female rats, indicated by lower levels of freezing due to pre-exposure to the cue. Estrous cycle did not affect latent inhibition at any age.

**Discussion:**

These results suggest that benign experience to a cue reduces subsequent conditioning to the cue more potently in adolescence compared to adulthood, which suggests a potential resilience mechanism naturally occurring in adolescence.

## 1 Introduction

Latent inhibition is when prior benign experience with a cue can impede or inhibit subsequent emotional learning to the cue (Lubow, 1973; Mineka and Zinbarg, 2006). In Pavlovian conditioning, latent inhibition is observed when the pre-exposure to the conditioned stimulus (CS) interferes with acquisition (George et al., 2010; Shalev et al., 1998; Lubow and Moore, 1959) or retrieval (Leung et al., 2013; Lingawi et al., 2016) of CS association with the unconditioned stimulus, measured by decreased conditioned responding in pre-exposed compared to non-pre-exposed subjects. Latent inhibition is proposed to explain how prior benign experiences prevent the development of experience-based anxiety disorders (Mineka and Zinbarg, 2006). For example, children who did not initially experience stress with dentists are much less likely to develop dental phobias following a subsequent traumatic experience (Davey, 1989). Similarly, monkeys who have observed models playing with snakes in seemingly safe circumstances do not develop fearful behaviors when directly exposed to snakes (Mineka and Cook, 1986).

Adolescence is a period of elevated risk to anxiety disorders (Polanczyk et al., 2015), which may be associated with latent inhibition. Indeed, anxiety symptoms in humans are related to deficits in inhibitory learning and memory measured by extinction of conditioned fear (Ganella et al., 2018a; Marin et al., 2017). Like latent inhibition, extinction involves benign exposures to a cue, except that such exposure occurs after the emotional learning (Bouton, 1993). Adolescents have been shown to display extinction impairments compared to adults in rats and humans (Ganella et al., 2017a,2018a; Ganella and Kim, 2014; Pattwell et al., 2012, 2013; Zbukvic et al., 2017). One study has shown that postnatal day 35 (P35) adolescent male rats indeed show latent inhibition, as measured by conditioned suppression of licking behavior in response to a tone CS that was paired with shocks (Zuckerman et al., 2003). However, latent inhibition has not yet been directly compared between adolescents and adults, which is the first aim of the present study. We hypothesize that the behavior of adolescents and adults will be differentially affected by latent inhibition because vulnerability towards anxiety disorders in adolescence (McGrath et al., 2023) has long been associated with the reduced function of medial prefrontal cortex (mPFC), which undergoes dramatic changes in structure and neurochemistry across adolescence (Casey et al., 2008; Kim et al., 2017; Perry et al., 2021; Gold et al., 2020). Notably, previous literature in rats gives rise to opposing predictions. On the one hand, temporary inactivation of the infralimbic cortex (IL) of mPFC led to deficits in latent inhibition (Lingawi et al., 2016, 2018), which suggests that latent inhibition would be impaired in adolescence. On the other, IL lesions facilitated latent inhibition (George et al., 2010), which suggests that latent inhibition would be facilitated in adolescents. Moreover, mPFC lesions did not affect latent inhibition in other studies (Joel et al., 1997; Lacroix et al., 1998) suggesting that latent inhibition may be comparable in adolescent and adult rats.

The second aim of the present study is to assess latent inhibition in males and females. Biological sex is a significant factor for anxiety disorders, with greater prevalence reported within the female population (Kessler et al., 2005). Sex differences in inhibitory learning and memory have been observed (Milad et al., 2009), although this effect is strongly mediated by hormonal effects/estrous cycle in female rodents and humans (Lebron-Milad et al., 2012; Graham and Milad, 2013). Interestingly, these estrous cycle effects are age dependent. Stages where there are high levels of estradiol are associated with improved extinction in adult females (Lebron-Milad et al., 2012; Graham and Milad, 2013), and impaired extinction in adolescent females (Perry et al., 2020). In addition, sex effects in extinction have been associated with differences in mPFC function (Day et al., 2020). Hence, latent inhibition may also be sex-specific. To our knowledge, there have been no studies that have examined latent inhibition of conditioned fear in female rodents. We hypothesize females will show age-specific effects of estrous cycle on latent inhibition. Specifically, latent inhibition would be greater when adult rats are in proestrous (i.e., when estradiol levels are high), while the opposite may be true in adolescent rats.

## 2 Materials and methods

### 2.1 Subjects

Male (26 adults and 22 adolescents) and female (44 adults and 37 adolescents) Sprague Dawley rats were bred in-house at the Florey Institute of Neuroscience and Mental Health. All rats were weaned at P21 and housed for the remainder of experimental procedures in groups of 3–6, with same-sex littermates in individually ventilated cages under a 12/12 h cycle (lights on at 7 a.m.) with food and water available *ad libitum*. All behavioral testing occurred during the light phase. On the first day of behavioral experimentation, rats in the adolescent groups were P35 (± 1), while rats in the adult groups were P70–P98. All rats were handled three times prior to commencement of behavioral experimentation. All procedures were approved by the Animal Care and Ethics Committee at the Florey Institute of Neuroscience and Mental Health in accordance with the guidelines for animal use set out in the *Australian Code of Practice for the Care and Use of Animals for Scientific Purposes* (8^th^ edition, 2013).

### 2.2 Apparatus

All behavior occurred in Contextual Near Infra-Red Fear Conditioning and Video Freeze system (Med Associates, VT, United States), which provides scrambled direct current footshocks. The dimensions of the chambers and the grid floor were as described previously (Zbukvic et al., 2017). A white house light was on during all sessions, and chambers were cleaned with 80% v/v ethanol and dried thoroughly between tests with rats being placed 5 min after drying.

### 2.3 Procedure

#### 2.3.1 Pre-exposure

On day 1, all rats were placed in the novel chambers. Following a 2-min baseline period, rats in group “pre-exposure” were presented with 45 tones (5,000 Hz, 80 dB), which served as the conditioned stimulus (CS). Each CS presentation was 10 s, with an inter-trial interval (ITI) of 10 s with 2-min post-tone period followed by the rats being returned to their home cages, which is standard in our laboratory for the first day of behavior in developing rats (Park et al., 2020; Luikinga et al., 2019). Such protocol was developed to allow some time to adjust at the end of the first behavioral session. Rats in group “No pre-exposure” were placed in the chambers for the same length of time (19 min), however, no tones were delivered.

#### 2.3.2 Conditioning

On day 2, all rats were placed in the same chamber as the previous day. We used a fear conditioning protocol that produces robust freezing to the tone that persists to subsequent sessions in developing and adult rats (Ganella et al., 2017b,2018b; Park et al., 2017). Following a 2 min baseline period the CS was presented for 10 s, co-terminating with a 1 s footshock (the unconditioned stimulus, US). This CS-US pairing was repeated three times with an ITI averaging 110 s, with 2-min post-shock period followed by the rats being returned to their home cages, which is standard in our laboratory to allow some time to adjust after the shock (Park et al., 2020; Luikinga et al., 2019). The intensity of the shock was different between Experiment 1 (males) and Experiment 2 (females). For males, the US was a 0.45 mA shock. Pilot data from the laboratory showed that this intensity produced robust freezing and reliable latent inhibition. However, for female rats, the US was 0.6 mA because pilot data showed that a 0.45 mA shock failed to produce robust freezing across conditioning and at test.

#### 2.3.3 Test

On day 3, all rats were placed in the same chamber as the previous day. As for the previous sessions, there was a 2-min baseline period after which the 10 s CS was presented 20 times in the absence of any footshock. ITI was 10 s.

#### 2.3.4 Estrous phase monitoring

For Experiment 2, vaginal lavages were taken from female rats 1 h after each behavioral session as described previously (Perry et al., 2020). Briefly, a pipette tip containing 20 μL of saline was used to flush the vagina two or three times if the vagina was opened. The fluid collected was dried on a microscope slide then stained with a 4% (v/v) methylene blue solution. Slides were then rinsed twice with distilled water, air dried, and observed under a light microscope (10 × magnification, Olympus BH-2). Slides were cross-checked by two independent researchers in a double-blind manner. They were categorized according to the proestrus, estrus, metestrus, and diestrus cycle using the morphology of cells and their relative cellular proportions (Cora et al., 2015). Rats in diestrus and metestrus were pooled together for statistical analyses as reported previously (Perry et al., 2020; Hecht et al., 1999), due to their similarities in rising levels of estrogen and the relatively short length of metestrus (Westwood, 2008).

### 2.4 Data analysis

All analyses were carried out using SPSS (IBM Corp., NY, United States). Percent freezing from all behavioral sessions was subjected to analysis of variance (ANOVA) or repeated-measures (RM) ANOVA. *Post hoc* analyses of simple effects using the Bonferroni correction were used to explain significant interactions wherever appropriate (Drummond et al., 2021). For analysis of CS-elicited freezing during pre-exposure, percent freezing across CS trials were averaged. For group No pre-exposure that did not receive any CS trials during the pre-exposure session, freezing was calculated for the matching periods as the group Pre-exposure. For analysis of CS-elicited freezing during conditioning, the first 9 s of each CS-US trial was used to discount the effects of shock on movement during the last second of each CS-US trial. Freezing during test was blocked into five CS presentations to result in four blocks. For all analyses in females, estrous phase at each behavioral session was included as a factor to account for potential hormonal effects. Metestrus and diestrus were pooled into a single group due to the shortness of metestrus phase as reportedly previously (Perry et al., 2020). Speed measurements of the rat center of mass were used to determine shock responsivity to the US and occurrences of darting to the CS (Carroll et al., 2025; Mitchell et al., 2024). Using DeepLabCut [version 3.0.0rc10; (Mathis et al., 2018; Nath et al., 2019)], 200 frames from ∼90% randomly selected videos from conditioning and test were assigned a tracking point to the rat center of mass. Using these annotated frames, the DeepLabCut markerless tracking model was generated using the ResNet50 neural network for 200 epochs using the PyTorch engine. All videos from conditioning and test were analyzed with this model then fed into the Simple Behavioral Analysis software [SimBA; (Goodwin et al., 2024)] to calculate speed (cm/s). Shock sensitivity was calculated as speed of movement during 1 s shock averaged across trials. Darting was defined as movement across the chamber at or exceeding 20 cm/s during the CS as previously described (Mitchell et al., 2024).

## 3 Results

### 3.1 Baseline freezing

**FIGURE 1:**
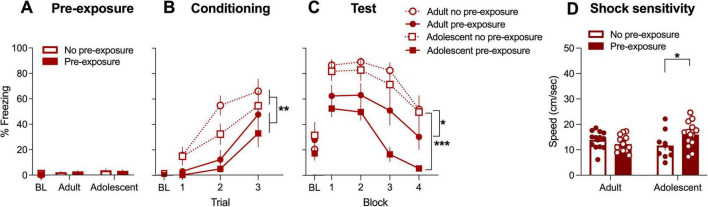
Baseline and CS-elicited freezing across **(A)** pre-exposure, **(B)** conditioning and **(C)** test sessions and **(D)** shock sensitivity in males. During conditioning, pre-exposed rats showed lower freezing compared to non-pre-exposed rats (main effect of Pre-exposure **p < 0.001), however, there were no differences between adults and adolescents. At test, both adults and adolescents in the pre-exposed condition showed lower freezing compared to non-pre-exposed counterparts, however, this effect was greater for adolescents (significant Age × Pre-exposure interaction, post-hoc tests *p < 0.05, ***p < 0.0001). Pre-exposed adolescent rats showed greater shock sensitivity than non-pre-exposed adolescents (significant Age × Pre-exposure interaction, post-hoc tests *p < 0.05], whereas this effect was not evident in adult rats. BL, baseline.

**FIGURE 2:**
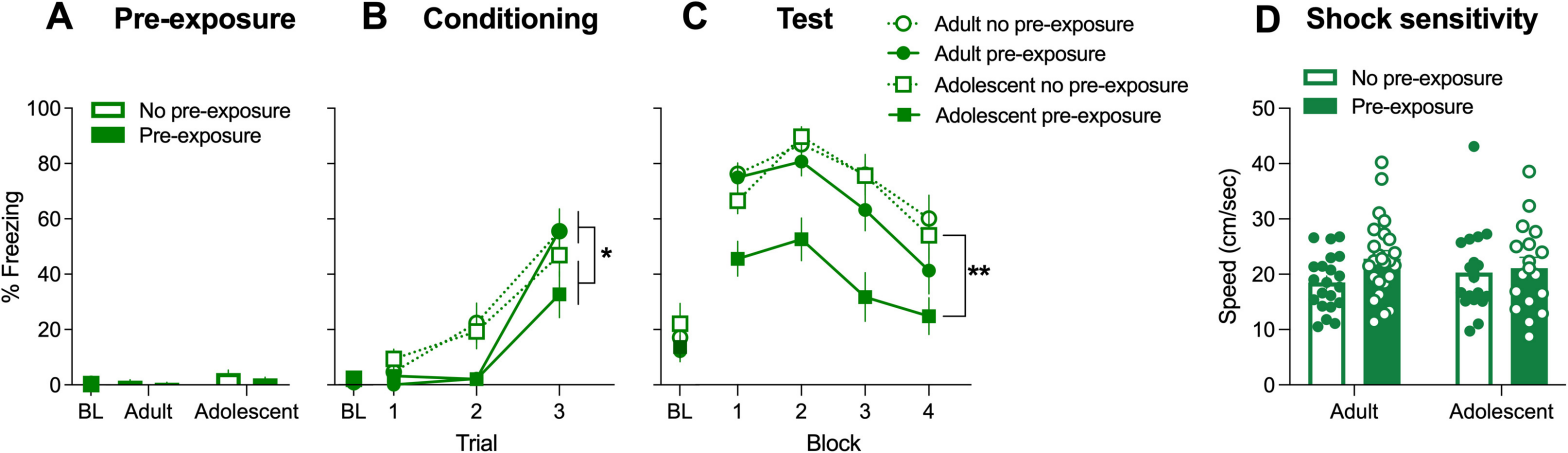
Baseline and CS-elicited freezing across **(A)** pre-exposure, **(B)** conditioning and **(C)** test sessions and **(D)** shock sensitivity in females. During conditioning, pre-exposed rats showed lower freezing compared to non-pre-exposed rats (main effect of Pre-exposure *p < 0.05), however, there were no differences between adults and adolescents. At test, only adolescents in the pre-exposed condition showed lower freezing compared to Non pre-exposed counterparts (significant Age × Pre-exposure interaction, post-hoc tests, **p < 0.05). There were no group differences in shock sensitivity. BL, baseline.

Freezing was recorded during the 2-min baseline periods for all sessions, which was clearly low during each session (Figures 1, 2 and Table 1). However, 2-way ANOVAs (Age × Pre-exposure) revealed significant main effects of Age during the conditioning baseline period for Experiment 1 [F(1,77) = 4.85, *p* = 0.03] and Experiment 2 [F(1,77) = 6.58, *p* = 0.01], with adolescents freezing more than adults in both experiments. Neither the Pre-exposure main effect nor the Age × Pre-exposure interaction were significant in any of the three sessions in any experiment (*p*’s > 0.05). In fact, prior to experiencing the first shock (i.e., across Pre-exposure session and Conditioning baseline), freezing in both sexes was very rarely observed (Table 1). During baseline, most rats showed 0% freezing. As such, the significant main effect of Age appears to be driven by the lack of variability and are unlikely to be meaningful.

**TABLE 1:** Mean (SEM) baseline freezing expressed as a percentage of total baseline period each behavioral session.

	Pre-exposure	Conditioning	Test
**Males**			
Adult: no pre-exposure	0.3 (0.2)	1.6 (0.6)	20.3 (7.4)
Adult: pre-exposure	0.3 (0.1)	1.3 (0.5)	27.8 (8.8)
Adolescent: no pre-exposure	1.2 (0.5)	1.7 (0.5)	31.4 (10.9)
Adolescent: pre-exposure	0.5 (0.3)	0.6 (0.3)	17.2 (5.7)
**Females**			
Adult: no pre-exposure	1.5 (0.3)	1.0 (0.5)	17.1 (6.3)
Adult: pre-exposure	0.8 (0.2)	0.5 (2.7)	12.2 (3.9)
Adolescent: no pre-exposure	4.3 (1.1)	2.2 (0.7)	22.2 (7.5)
Adolescent: pre-exposure	2.4 (0.4)	1.8 (0.5)	13.7 (4.3)

Note that there was a significant difference between adolescents and adults (averaged across pre-exposure condition i.e., main effect of Age) on the Conditioning day only (*p* < 0.5). However, this constitutes a difference of 0.3% freezing for males and 1.2% freezing for females, which is negligible compared to freezing levels once shock has been experienced, and is unlikely to constitute a biologically meaningful difference.

### 3.2 Adolescent males show greater propensity for latent inhibition compared to adult males

Freezing during pre-exposure session was low, regardless of whether rats were presented with the CS or not (Figure 1A). Two-way ANOVA (Age × Pre-exposure) of freezing during pre-exposure confirmed no main effect of Pre-exposure and no Age × Pre-exposure interaction (*p*’s > 0.05) indicating that the pre-exposure to the CS does not trigger freezing in adolescent and adult male rats. There was also no main effect of Age (*p* > 0.05).

Conditioned stimulus-elicited freezing during conditioning (Figure 1B) was analyzed using repeated measures ANOVA (Age × Pre-exposure x Conditioning trial). Mauchley’s Test of Sphericity indicated that the assumption of sphericity had been violated (*p* < 0.001), and therefore a Greenhouse-Geisser correction was applied to all repeated measures analyses. There were significant main effects of Conditioning trial [F(2, 44) = 37.33, *p* < 0.001] and Pre-exposure [F(1, 44) = 16.80, *p* < 0.001]. The main effect of Age was not significant [F(1,44) = 3.18, *p* = 0.08]. None of the two-way interactions, nor the three-way interactions were significant (*p*’s > 0.05). Thus, all rats significantly increased CS-elicited freezing across the conditioning trials, with pre-exposed groups overall freezing less than no pre-exposure groups regardless of age.

Figure 1C shows freezing during the 20 CS presentations at test, binned into blocks of five CS presentations. Three-way ANOVA (Age × Pre-exposure x Block) revealed significant main effects of Age [F(1,44) = 4.865, *p* < 0.05], Pre-exposure [F(1,44) = 38.983, *p* < 0.001] and Block [F(3,44) = 69.25, *p* < 0.001]. There was a significant Age × Pre-exposure interaction [F(1,44) = 11.87, *p* = 0.001], and although Block × Pre-exposure interaction was significant [F(3,44) = 3.23, *p* < 0.05], Age × Block × Pre-exposure was not (*p* > 0.05). The post hoc analysis revealed that although latent inhibition was expressed for both adults and adolescents (i.e., there was a significant difference between pre-exposed and non-pre-exposed rats), this effect was greater for adolescents compared to adults [Adults: 95% CI (0.40, 30.76); Adolescents; 95% CI (37.42, 70.47)]. Furthermore, freezing for the pre-exposed rats was greater than non-pre-exposed rats at all four blocks (all *p*’s < 0.001). Taken together, adolescent male rats showed greater latent inhibition following a protocol than adult males, and reduced freezing at test was due reduced retrieval of fear in pre-exposed rats that was apparent from the first block of CS presentations.

Shock sensitivity averaged across three shock exposures (Figure 1D) was analyzed using two way ANOVA (Age × Pre-exposure). There were no main effects for Age nor Pre-exposure [F(1,44) = 0.35 and 1.13, respectively, *p*’s > 0.05], however, there was a significant Age × Pre-exposure interaction [F(1,44) = 5.38, *p* < 0.05]. Bonferroni-corrected simple effects revealed greater sensitivity to shock in pre-exposed compared to non-pre-exposed adolescent (*p* < 0.05), but not adult (*p* = 0.353). This indicates that the interaction was driven by increased shock sensitivity in pre-exposed group for adolescents only. Darting was negligible across both conditioning and test (only a single dart of moving across the chamber in all the groups was recorded during conditioning and test). Therefore, this behavior could not be analyzed.

### 3.3 Adolescent females show propensity for latent inhibition compared to adult females

Three-way ANOVA of freezing during pre-exposure showed no significant main effects nor interactions of Age, Pre-exposure, and Estrous (*p*’s > 0.05). These results suggest that the pre-exposure to the CS and different estrous phases do not elicit freezing in adolescent and adult female rats (Figure 2A).

Conditioned stimulus-elicited freezing during conditioning (Figure 2B) was analyzed using repeated measures ANOVA (Age × Pre-exposure × Estrous × Conditioning Trial). Mauchley’s Test of Sphericity indicated that the assumption of sphericity had been violated (*p* < 0.001), and therefore a Greenhouse-Geisser correction was applied to all repeated measures analyses. Similarly, to males, there was a main effect of Conditioning trial [F(2,136) = 73.23, *p* < 0.001], indicating freezing increased across repeated trials. There also was a main effect of Pre-exposure [F(1,68) = 8.28, *p* = 0.005], showing that freezing across the three CS presentations was overall lower following pre-exposure to the stimulus. Main effects of Age and Estrous phase were non-significant [F(1,68) = 1.34, *p* = 0.25; F(3,67) = 0.24, *p* = 0.87, respectively]. There were no two, three nor four-way significant interactions (*p*’s > 0.05).

**FIGURE 3:**
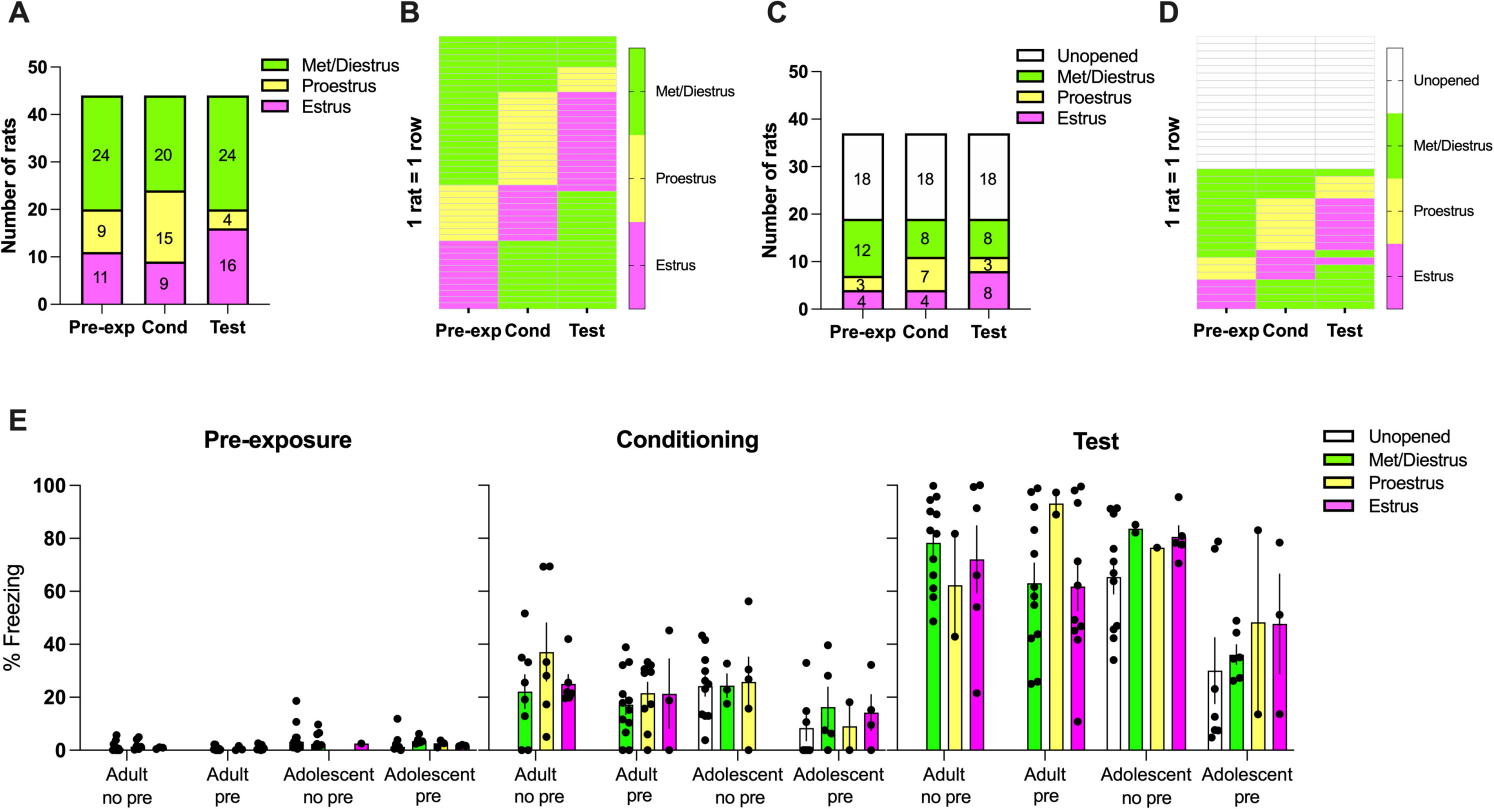
Estrous cycle information for adult and adolescent females. The number of **(A)** adult and **(C)** adolescent rats in each estrous phase on pre-exposure, conditioning and test days. Progression of individual **(B)** adult and **(D)** adolescent rats in estrous phase across experimental days. Each row represents one rat. **(E)** Average freezing on each day with respect to group and estrous phase on that day. Pre, pre-exposure, No pre, no pre-exposure.

Figure 3C shows freezing during the 20 CS presentations at test, binned into blocks of five CS presentations. Four-way (Age × Pre-exposure × Block × Estrous) ANOVA revealed a main effect of Pre-exposure [F(1,68) = 12.052, *p* < 0.001] and Pre-exposure × Age interaction [F(1,68) = 5.00, *p* < 0.05]. Main effects of Age and Estrous were not significant [F(1,68) = 3.94, *p* = 0.051; F(3,68) = 1.15, *p* = 0.33, respectively]. Main effect of Block was significant [F(3,68) = 27.87, *p* < 0.001], however, there were no significant interactions involving Block (two, three, and four-way Block interaction *p*’s > 0.05). There were no significant interactions involving Estrous (*p*’s > 0.05). Since there were no interactions with Estrous, we determined the simple effects averaging across Estrous phase. These showed that there was no significant effect of Pre-exposure for adults [*p* = 0.86, 95% CI (−21.55, 17.99)], where there was for adolescent female rats [*p* < 0.001, 95% CI (17.77, 59.69)]. Therefore, as with male rats, the latent inhibition effect was greater in adolescent female rats when compared with adults. Lack of interaction with Block showed that this was due to reduced retrieval of fear in Adolescent pre-exposed rats from the first CS presentation.

Shock sensitivity averaged across three shock exposures (Figure 2D) was analyzed using two-way ANOVA (Age × Pre-exposure). There were no main effects for Age nor Pre-exposure [F(1,68) = 0.01 and 2.55, respectively, *p*’s > 0.05], nor was there a significant Age × Pre-exposure interaction [F(1,68) = 1.17, *p* > 0.05]. Darting was not observed and could not be analyzed.

In order to verify that rats were cycling, we analyzed changes in proportion of rats in each phase across behavioral days as previously reported (Perry et al., 2020). As expected, adult females showed significant [χ2(4,44) = 11.07, *p* < 0.05] changes in the proportion of estrus phases (Figure 3A) whereas adolescent females did not [χ2(6,37) = 5.60, *p* = 0.47] (Figure 3C). Examination of individual rats revealed that many adolescent rats have not begun estrus cycling (Figure 3D). Interestingly, the adolescents that have begun cycling and adults largely showed similar expected pattern of 2–3 days met/diestrus, ∼1 day of proestrus and ∼1 day of estrus (Figures 3B, D), unlike the previous study in which adolescents showed much more erratic cycling (Perry et al., 2020). Average freezing levels were graphed for each phase (Figure 3D). We could not analyze the data (even with excluding unopened groups) because there were 0–1 rats for some phases in each session. There appears to be very little impact of estrous phase within groups.

## 4 Discussion

The aim of this study was to examine age differences (adolescent vs. adult) in latent inhibition in male and female rats. We found that adolescents were more likely to display evidence of latent inhibition when compared to adults, with lower levels of freezing shown in adolescents compared to adults in the pre-exposed rats. These findings do not exclude that latent inhibition occurs for adults as well, since there is evidence of delayed conditioning in the pre-exposed rats on day 2 regardless of age and sex (Figures 1, 3). This delayed learning was not due to decreased sensitivity to the shock as a result of pre-exposure to the CS. If anything, pre-exposed animals showed greater sensitivity, although this effect was only evident in adolescent males. Thus, during pre-exposure both adults and adolescents had learned that the CS has no aversive consequence, and this learning delays acquisition of the CS-shock association similarly across age groups. However, the effect of the initial experience is clearly more enduring for adolescents, since pre-exposure was more likely to reduce fear expression on day 3 in that age group. Surprisingly, despite previous studies that fear learning is affected by sex hormones (Graham and Milad, 2013, 2014; Perry et al., 2020), we found no effect of estrous in our female rats.

The enhanced latent inhibition observed here in adolescents is similar to effects seen following experimental lesion of the IL (George et al., 2010). Compared to other brain regions, the mPFC is last to reach maturity in rodents and humans (Casey et al., 2008; Kim et al., 2017; Gogtay et al., 2004; Giedd, 2004; Bjerke et al., 2025). It is therefore possible that the enhanced latent inhibition effect observed here in adolescents may be due to reduced functionality in the IL mPFC, which led to stronger effects of pre-exposure. When IL lesions facilitated latent inhibition, George et al. (2010) argued that the role of IL is to allow the learning of a second, conflicting information about a cue. As such, IL hypofunction during adolescence results in enhanced latent inhibition because the second association (cue-shock) is not encoded properly. Such a role for IL is also consistent with previous studies showing impaired extinction and reduced IL function in adolescents (Ganella et al., 2018a; Kim et al., 2011). In contrast, others have shown that temporary inactivation of the IL inhibited retrieval of latent inhibition (Lingawi et al., 2016, 2018). The differences in findings may be explained by specific methodology. In George et al. (2010), IL lesions occurred prior to any behavioral training. Thus, function was impaired across pre-exposure and at test. When IL inactivation only occurred immediately prior to retrieval test (Lingawi et al., 2016, 2018), IL function would have been intact during pre- exposure. From this, IL may be critical for remembering the latent inhibition memory once pre-exposure and conditioning have already occurred. In our study, assumed IL hypofrontality was due to developmental stage rather than experimental manipulation, hence would have been present during pre-exposure and conditioning as with George et al. (2010).

Importantly, brain maturation is not restricted to the IL and the present results may be due to dopamine signaling in the nucleus accumbens (NAc). A recent study showed that cue-elicited dopamine release in the NAc core was directly linked to latent inhibition (Kutlu et al., 2022). Specifically, exposure to the cue evokes dopamine release, which declines with repeated exposures across the pre-exposure session. Therefore, during conditioning, the pre-exposed cue evokes a smaller dopamine release than the non-pre-exposed cue, meaning that it is less able to form an association with the US (Kutlu et al., 2022). At conditioning, intra-nucleus accumbens (NAc) injections of amphetamine attenuated, while a dopamine receptor 2 (D2) antagonist (haloperidol) enhanced latent inhibition (Joseph et al., 2000). However, intra-mPFC injection of amphetamine or apomorphine had no effects (Lacroix et al., 2000; Broersen et al., 1999). Furthermore, deficits in latent inhibition of conditioned fear due to adolescent social isolation were associated with increased D2 expression in the mPFC and NAc (Han et al., 2012). Conversely, a dopamine receptor 1 (D1) agonist administered systemically prior to pre-exposure and conditioning sessions mildly inhibited latent inhibition of fear conditioning (Feldon et al., 1991). A D1 antagonist (SCH23390) administered alone had no effect, but did block amphetamine-induced disruption of latent inhibition (Nelson et al., 2012). In summary, attenuated D1 or D2 signaling appears to promote latent inhibition, which can explain the present age differences observed with D1 reduced in the ventral striatum but not mPFC in adolescence compared to adulthood (Bjerke et al., 2025; Cullity et al., 2019). Future studies should look at differential effects of D1 and D2 receptor modulators in adults and adolescents in order to better understand these effects.

Latent inhibition involves targeted redirection of attention following pre-exposure to the stimulus (Colagiuri et al., 2021; Lubow, 2005). In other words, there is decreased attention directed towards to the target stimulus following pre-exposure, making it less available for association to the US in subsequent conditioning sessions. Children with attention deficit hyperactivity disorder showed reduced latent inhibition compared to controls in a discrimination task (Lubow and Josman, 1993) and a visual search task (Lubow, 2005). Within this context, the current findings suggest that adolescents were more able to attend to non-predictive stimuli than adults. However, we also observed that both adults and adolescents acquired conditioned fear to the non-pre-exposed stimulus, meaning that both ages are equally able to attend to a stimulus when it is novel. Further, the age difference in latent inhibition only emerged at test, which suggests a retrieval rather than an encoding effect. Nevertheless, this does not rule out that a weaker association was formed between the pre-exposed CS and the US due to increased attention in the adolescent group.

We did not find any effects for estrous cycle during conditioning or at test. In contrast, extinction is impacted by the estrous phase (Milad et al., 2009; Perry et al., 2020), and by the experimental manipulation of gonadal hormones (Perry et al., 2020; Zeidan et al., 2011; Maeng et al., 2017). Interestingly, this effect on extinction is also apparently age-dependent, because in adults high levels of estrogen facilitate extinction (Milad et al., 2009; Maeng and Milad, 2015; Velasco et al., 2019), while in adolescents extinction is facilitated where estrogen is not present/present at low levels (Perry et al., 2020). In the current study there were no differences in latent inhibition reported where rats were at different stages of estrous cycle, regardless of age. This suggests that interference between a benign memory and a traumatic experience is not dependent on cycling gonadal hormones and implies that there are distinct neural mechanisms underlying extinction and latent inhibition. Notably, the present study observed overall reduced variation in estrous cycling in adolescents due to many animals staying unopened throughout the behavioral days, which is different from the previous study in which the adolescent female rats of the same age were much more variable in their estrus phases (Perry et al., 2020). It is possible that testing adults and adolescents together as in the present study affected estrous cycling, as previous studies have shown female rats in proximity can affect their estrous phase (Alekhina et al., 2015). Different outcomes may be observed if the adolescent and adult females are not concurrently tested.

A limitation of this study is that male and female cohorts were run in separate experiments, meaning that direct statements regarding sex differences cannot be made. We chose to treat the two cohorts as separate experiments due to differences in shock sensitivity. Specifically, male rats showed robust freezing and latent inhibition effect (at least for adolescents) using a 0.45 mA shock, while for females freezing was unreliable using these parameters, and the shock needed to be increased to 0.6 mA in order to obtain reliable conditioning. Sex differences in sensitivity to footshock and consequent conditioning have been observed previously (Stock et al., 2001; Gupta et al., 2001; Wiltgen et al., 2001; Baran et al., 2009), although this is not always the case (Orsini et al., 2016). Sex differences in latent inhibition have been reported for a conditioned taste aversion whereby adult female rats showed weaker latent inhibition than males (Angulo et al., 2020; Angulo and Arévalo-Romero, 2021). Interestingly, the same study saw delayed extinction in males compared with females (Angulo and Arévalo-Romero, 2021), which is different from what we (Perry et al., 2020) and others (Velasco et al., 2019) have observed. Furthermore, human participants did not show sex differences in latent inhibition for a discrimination task (Lubow and Josman, 1993). Indeed, it is possible that sex specificity of latent inhibition is task-dependent.

Notably, all sessions occurred in the same context in the present study, which means that latent inhibition of context conditioning may have occurred (Killcross et al., 1998; Westbrook et al., 1994; Kiernan and Westbrook, 1993). That is, prolonged pre-exposure to a context can reduce subsequent conditioning in that context. However, typical latent inhibition of contextual conditioned fear involves extensive pre-exposure to the context, such as 20 min twice a day for 11 days (Killcross et al., 1998). Interestingly, shorter exposure to the context can lead to “context pre-exposure facilitation effect” (Heroux et al., 2018; Fanselow, 1986; Robinson-Drummer and Stanton, 2015), which is increased context conditioned fear when there is a presentation of the footshock as soon as the rodent is placed into the context. Without context pre-exposure, such immediate shock in a context leads to a failure in context conditioning (“immediate shock deficit”) (Fanselow, 1986; Robinson-Drummer and Stanton, 2015). Such deficit is not so apparent with discrete cues, highlighting how forming a contextual representation requires longer time than forming a discrete cue representation (Rudy, 1993; Kim and Richardson, 2009). This may be due to the necessity of the different elements in the context to come together to form a spatial representation in contextual learning (Rudy and Sutherland, 1995). In any case, with the inclusion of “No pre-exposure” group that received an identical amount of context exposure as the pre-exposed group, it is unlikely that the present age effects are due to differences in latent inhibition of contextual conditioned fear.

In summary, the current findings show that adolescents show stronger latent inhibition than adults. Latent inhibition serves an adaptive purpose (Lubow, 2005). For example, it helps to focus attention away from stimuli less likely to be relevant in a new situation, thereby preserving cognitive load. It may also prevent too much predictive value being attributed to stimuli that are only salient in passing/in certain contexts. Thus, this facilitated latent inhibition in adolescents reflects experience-dependent resilience as they are able to form robust CS-no-event association that is more readily retrieved following conditioning when the rats are tested in extinction (i.e., safe) conditions (Mineka and Zinbarg, 2006). The fact that age-differences in expression of latent inhibition did not emerge until test (under extinction conditions) provides weight to this interpretation. However, future studies should explore this possibility among others, such as reduced behavioral flexibility and changes in attention, in order to provide a more comprehensive understanding on how learning styles change across development. Anxiety disorders often emerge in adolescence, and this has led to the perspective that adolescents are more vulnerable (Polanczyk et al., 2015; Ganella et al., 2018a; Kim and Ganella, 2015). The current findings show that this story is more complex, because facilitated latent inhibition may provide resilience against development of anxiety. Notably, this resilience is dependent on experiential factors, since latent inhibition is dependent on prior exposure to the stimuli. In other words, timing of traumatic experiences relative to other experiences across adolescence is critical for determining how much of an impact the trauma will have on subsequent behavior.

## Data Availability

The raw data supporting the conclusions of this article will be made available by the authors, without undue reservation.

## References

[B1] Alekhina T. Klochkov D. Pal’chikova N. Kuz’minova O. Prokudina O. (2015). Synchronization of the estrous cycle against the background of increased excitability in rats selected for catatonic type of reaction. *Bull. Exp. Biol. Med.* 159 73–76. 10.1007/s10517-015-2893-x 26033595

[B2] Angulo R. Arévalo-Romero C. (2021). Sexual dimorphism in classical conditioning? Sex differences in neophobia, latent inhibition, generalization, and extinction for rats (*Rattus norvegicus*) in a conditioned taste aversion preparation irrespective of housing conditions. *J. Comp. Psychol.* 135 315–326. 10.1037/com0000275 34553981

[B3] Angulo R. Bustamante J. Arévalo-Romero C. (2020). Age, sex and pre-exposure effects on acquisition and generalization of conditioned taste aversion in rats. *Behav. Brain Res.* 394:112813. 10.1016/j.bbr.2020.112813 32712137

[B4] Baran S. Armstrong C. Niren D. Hanna J. Conrad C. (2009). Chronic stress and sex differences on the recall of fear conditioning and extinction. *Neurobiol. Learn. Mem.* 91 323–332. 10.1016/j.nlm.2008.11.005 19073269 PMC2673234

[B5] Bjerke I. Carey H. Bjaalie J. Leergaard T. Kim J. (2025). The developing mouse dopaminergic system: Cortical-subcortical shift in D1/D2 receptor balance and increasing regional differentiation. *Neurochem. Int.* 182:105899. 10.1016/j.neuint.2024.105899 39537102

[B6] Bouton M. (1993). Context, time, and memory retrieval in the interference paradigms of Pavlovian learning. *Psychol. Bull.* 114 80–99. 10.1037/0033-2909.114.1.80 8346330

[B7] Broersen L. Feldon J. Weiner I. (1999). Dissociative effects of apomorphine infusions into the medial prefrontal cortex of rats on latent inhibition, prepulse inhibition and amphetamine-induced locomotion. *Neuroscience* 94 39–46. 10.1016/s0306-4522(99)00287-0 10613495

[B8] Carroll J. Myers B. Vaaga C. (2025). Repeated presentation of visual threats drives innate fear habituation and is modulated by threat history and acute stress exposure. *Stress* 28:2489942. 10.1080/10253890.2025.2489942 40219787 PMC12065417

[B9] Casey B. Jones R. Hare T. (2008). The adolescent brain. *Ann. N. Y. Acad. Sci.* 1124 111–126. 10.1196/annals.1440.010 18400927 PMC2475802

[B10] Colagiuri B. Park J. Barnes K. Sharpe L. Boakes R. Colloca L. (2021). Pre-exposure, but not overshadowing, inhibits nocebo hyperalgesia. *J. Pain* 22 864–877. 10.1016/j.jpain.2021.02.008 33636369

[B11] Cora M. Kooistra L. Travlos G. (2015). Vaginal cytology of the laboratory rat and mouse: Review and criteria for the staging of the estrous cycle using stained vaginal smears. *Toxicol. Pathol.* 43 776–793. 10.1177/0192623315570339 25739587 PMC11504324

[B12] Cullity E. Madsen H. Perry C. Kim J. (2019). Postnatal developmental trajectory of dopamine receptor 1 and 2 expression in cortical and striatal brain regions. *J. Comp. Neurol.* 527 1039–1055. 10.1002/cne.24574 30408161

[B13] Davey G. (1989). Dental phobias and anxieties: Evidence for conditioning processes in the acquisition and modulation of a learned fear. *Behav. Res. Ther.* 27 51–58. 10.1016/0005-7967(89)90119-8 2914005

[B14] Day H. Suwansawang S. Halliday D. Stevenson C. (2020). Sex differences in auditory fear discrimination are associated with altered medial prefrontal cortex function. *Sci. Rep.* 10:6300. 10.1038/s41598-020-63405-w 32286467 PMC7156682

[B15] Drummond K. Waring M. Faulkner G. Blewitt M. Perry C. Kim J. (2021). Hippocampal neurogenesis mediates sex-specific effects of social isolation and exercise on fear extinction in adolescence. *Neurobiol. Stress* 15:100367. 10.1016/j.ynstr.2021.100367 34337114 PMC8313755

[B16] Fanselow M. (1986). Associative vs topographical accounts of the immediate shock-freezing deficit in rats: Implications for the response selection rules governing species-specific defensive reactions. *Learn. Motiv.* 17 16–39. 10.1016/0023-9690(86)90018-4

[B17] Feldon J. Shofel A. Weiner I. (1991). Latent inhibition is unaffected by direct dopamine agonists. *Pharmacol. Biochem. Behav.* 38 309–314. 10.1016/0091-3057(91)90283-8 1676170

[B18] Ganella D. Kim J. (2014). Developmental rodent models of fear and anxiety: From neurobiology to pharmacology. *Br. J. Pharmacol.* 171 4556–4574. 10.1111/bph.12643 24527726 PMC4209932

[B19] Ganella D. Barendse M. Kim J. Whittle S. (2017a). Prefrontal-amygdala connectivity and state anxiety during fear extinction recall in adolescents. *Front. Hum. Neurosci.* 11:587. 10.3389/fnhum.2017.00587 29255411 PMC5722839

[B20] Ganella D. Drummond K. Ganella E. Whittle S. Kim J. (2018a). Extinction of conditioned fear in adolescents and adults: A human fMRI study. *Front. Hum. Neurosci.* 11:647. 10.3389/fnhum.2017.00647 29358913 PMC5766664

[B21] Ganella D. Lee-Kardashyan L. Luikinga S. Nguyen D. Madsen H. Zbukvic I. (2017b). Aripiprazole facilitates extinction of conditioned fear in adolescent rats. *Front. Behav. Neurosci.* 11:76. 10.3389/fnbeh.2017.00076 28536511 PMC5422437

[B22] Ganella D. Nguyen L. Lee-Kardashyan L. Kim L. Paolini A. Kim J. (2018b). Neurocircuitry of fear extinction in adult and juvenile rats. *Behav. Brain Res.* 351 161–167. 10.1016/j.bbr.2018.06.001 29898421

[B23] George D. Duffaud A. Pothuizen H. Haddon J. Killcross S. (2010). Lesions to the ventral, but not the dorsal, medial prefrontal cortex enhance latent inhibition. *Eur. J. Neurosci.* 31 1474–1482. 10.1111/j.1460-9568.2010.07178.x 20384772

[B24] Giedd J. (2004). Structural magnetic resonance imaging of the adolescent brain. *Ann. N. Y. Acad. Sci.* 1021 77–85. 10.1196/annals.1308.009 15251877

[B25] Gogtay N. Giedd J. Lusk L. Hayashi K. Greenstein D. Vaituzis A. (2004). Dynamic mapping of human cortical development during childhood through early adulthood. *Proc. Natl. Acad. Sci. U.S.A.* 101 8174–8179. 10.1073/pnas.0402680101 15148381 PMC419576

[B26] Gold A. Abend R. Britton J. Behrens B. Farber M. Ronkin E. (2020). Age differences in the neural correlates of anxiety disorders: An fMRI study of response to learned threat. *Am. J. Psychiatry* 177 454–463. 10.1176/appi.ajp.2019.19060650 32252541 PMC9078083

[B27] Goodwin N. Choong J. Hwang S. Pitts K. Bloom L. Islam A. (2024). Simple behavioral analysis (SimBA) as a platform for explainable machine learning in behavioral neuroscience. *Nat. Neurosci.* 27 1411–1424. 10.1038/s41593-024-01649-9 38778146 PMC11268425

[B28] Graham B. Milad M. (2013). Blockade of estrogen by hormonal contraceptives impairs fear extinction in female rats and women. *Biol. Psychiatry* 73 371–378. 10.1016/j.biopsych.2012.09.018 23158459 PMC3557577

[B29] Graham B. Milad M. (2014). Inhibition of estradiol synthesis impairs fear extinction in male rats. *Learn. Mem.* 21 347–350. 10.1101/lm.034926.114 24939838 PMC4061425

[B30] Gupta R. Sen S. Diepenhorst L. Rudick C. Maren S. (2001). Estrogen modulates sexually dimorphic contextual fear conditioning and hippocampal long-term potentiation (LTP) in rats(1). *Brain Res.* 888 356–365. 10.1016/s0006-8993(00)03116-4 11150498

[B31] Han X. Li N. Xue X. Shao F. Wang W. (2012). Early social isolation disrupts latent inhibition and increases dopamine D2 receptor expression in the medial prefrontal cortex and nucleus accumbens of adult rats. *Brain Res.* 1447 38–43. 10.1016/j.brainres.2012.01.058 22341870

[B32] Hecht G. Spear N. Spear L. (1999). Changes in progressive ratio responding for intravenous cocaine throughout the reproductive process in female rats. *Dev. Psychobiol.* 35 136–145. 10.1002/(SICI)1098-2302(199909)35:2<136::AID-DEV6>3.0.CO;2-K 10461127

[B33] Heroux N. Osborne B. Miller L. Kawan M. Buban K. Rosen J. (2018). Differential expression of the immediate early genes c-Fos, Arc, Egr-1, and Npas4 during long-term memory formation in the context preexposure facilitation effect (CPFE). *Neurobiol. Learn. Mem.* 147 128–138. 10.1016/j.nlm.2017.11.016 29222058 PMC6314028

[B34] Joel D. Weiner I. Feldon J. (1997). Electrolytic lesions of the medial prefrontal cortex in rats disrupt performance on an analog of the wisconsin card sorting test, but do not disrupt latent inhibition: Implications for animal models of schizophrenia. *Behav. Brain Res.* 85 187–201. 10.1016/s0166-4328(97)87583-3 9105575

[B35] Joseph M. Peters S. Moran P. Grigoryan G. Young A. Gray J. (2000). Modulation of latent inhibition in the rat by altered dopamine transmission in the nucleus accumbens at the time of conditioning. *Neuroscience* 101 921–930. 10.1016/s0306-4522(00)00437-1 11113341

[B36] Kessler R. Berglund P. Demler O. Jin R. Merikangas K. Walters E. (2005). Lifetime prevalence and age-of-onset distributions of DSM-IV disorders in the National Comorbidity Survey Replication. *Arch. Gen. Psychiatry* 62 593–602. 10.1001/archpsyc.62.6.593 15939837

[B37] Kiernan M. Westbrook R. (1993). Effects of exposure to a to-be-shocked environment upon the rat’s freezing response: Evidence for facilitation, latent inhibition, and perceptual learning. *Q. J. Exp. Psychol. B* 46 271–288. 10.1080/14640749308401089 8210452

[B38] Killcross A. Kiernan M. Dwyer D. Westbrook R. (1998). Effects of retention interval on latent inhibition and perceptual learning. *Q. J. Exp. Psychol. B* 51 59–74. 10.1080/713932665 9532962

[B39] Kim J. Ganella D. E. (2015). A review of preclinical studies to understand fear during adolescence. *Aust. Psychol.* 50 25–31. 10.1111/ap.12066

[B40] Kim J. Richardson R. (2009). Expression of renewal is dependent on the extinction-test interval rather than the acquisition-extinction interval. *Behav. Neurosci.* 123 641–649. 10.1037/a0015237 19485571

[B41] Kim J. Li S. Richardson R. (2011). Immunohistochemical analyses of long-term extinction of conditioned fear in adolescent rats. *Cereb. Cortex* 21 530–538. 10.1093/cercor/bhq116 20576926

[B42] Kim J. Perry C. Ganella D. Madsen H. (2017). Postnatal development of neurotransmitter systems and their relevance to extinction of conditioned fear. *Neurobiol. Learn. Mem.* 138 252–270. 10.1016/j.nlm.2016.10.018 27818267

[B43] Kutlu M. Zachry J. Melugin P. Tat J. Cajigas S. Isiktas A. (2022). Dopamine signaling in the nucleus accumbens core mediates latent inhibition. *Nat. Neurosci.* 25 1071–1081. 10.1038/s41593-022-01126-1 35902648 PMC9768922

[B44] Lacroix L. Broersen L. Feldon J. Weiner I. (2000). Effects of local infusions of dopaminergic drugs into the medial prefrontal cortex of rats on latent inhibition, prepulse inhibition and amphetamine induced activity. *Behav. Brain Res.* 107 111–121. 10.1016/s0166-4328(99)00118-7 10628735

[B45] Lacroix L. Broersen L. Weiner I. Feldon J. (1998). The effects of excitotoxic lesion of the medial prefrontal cortex on latent inhibition, prepulse inhibition, food hoarding, elevated plus maze, active avoidance and locomotor activity in the rat. *Neuroscience* 84 431–442. 10.1016/s0306-4522(97)00521-6 9539214

[B46] Lebron-Milad K. Abbs B. Milad M. Linnman C. Rougemount-Bücking A. Zeidan M. (2012). Sex differences in the neurobiology of fear conditioning and extinction: A preliminary fMRI study of shared sex differences with stress-arousal circuitry. *Biol. Mood Anxiety Disord.* 2:7. 10.1186/2045-5380-2-7 22738021 PMC3416700

[B47] Leung H. Killcross A. Westbrook R. F. (2013). A further assessment of the Hall-Rodriguez theory of latent inhibition. *J. Exp. Psychol. Anim. Behav. Process.* 39 117–125. 10.1037/a0031724 23586536

[B48] Lingawi N. Holmes N. Westbrook R. Laurent V. (2018). The infralimbic cortex encodes inhibition irrespective of motivational significance. *Neurobiol. Learn. Mem.* 150 64–74. 10.1016/j.nlm.2018.03.001 29518495

[B49] Lingawi N. Westbrook R. Laurent V. (2016). Extinction and latent inhibition involve a similar form of inhibitory learning that is stored in and retrieved from the infralimbic cortex. *Cereb. Cortex* 27 5547–5556. 10.1093/cercor/bhw322 27797830

[B50] Lubow R. (1973). Latent inhibition. *Psychol. Bull.* 79 398–407. 10.1037/h0034425 4575029

[B51] Lubow R. (2005). Construct validity of the animal latent inhibition model of selective attention deficits in schizophrenia. *Schizophr. Bull.* 31 139–153. 10.1093/schbul/sbi005 15888432

[B52] Lubow R. Josman Z. (1993). Latent inhibition deficits in hyperactive children. *J. Chil. Psychol. Psychiatry* 34 959–973. 10.1111/j.1469-7610.1993.tb01101.x 8408378

[B53] Lubow R. Moore A. (1959). Latent inhibition: The effect of nonreinforced pre-exposure to the conditional stimulus. *J. Comp. Physiol. Psychol.* 52 415–419. 10.1037/h0046700 14418647

[B54] Luikinga S. Perry C. Madsen H. Lawrence A. Kim J. (2019). Effects of methamphetamine exposure on fear learning and memory in adult and adolescent rats. *Neurochem. Res.* 44 2081–2091. 10.1007/s11064-019-02845-x 31338719

[B55] Maeng L. Milad M. (2015). Sex differences in anxiety disorders: Interactions between fear, stress, and gonadal hormones. *Horm. Behav.* 76 106–117. 10.1016/j.yhbeh.2015.04.002 25888456 PMC4823998

[B56] Maeng L. Taha M. Cover K. Glynn S. Murillo M. Lebron-Milad K. (2017). Acute gonadotropin-releasing hormone agonist treatment enhances extinction memory in male rats. *Psychoneuroendocrinology* 82 164–172. 10.1016/j.psyneuen.2017.05.015 28550793 PMC5596662

[B57] Marin M. Zsido R. Song H. Lasko N. Killgore W. Rauch S. (2017). Skin conductance responses and neural activations during fear conditioning and extinction recall across anxiety disorders. *JAMA Psychiatry* 74 622–631. 10.1001/jamapsychiatry.2017.0329 28403387 PMC5539837

[B58] Mathis A. Mamidanna P. Cury K. Abe T. Murthy V. Mathis M. (2018). DeepLabCut: Markerless pose estimation of user-defined body parts with deep learning. *Nat. Neurosci.* 21 1281–1289. 10.1038/s41593-018-0209-y 30127430

[B59] McGrath J. Al-Hamzawi A. Alonso J. Altwaijri Y. Andrade L. Bromet E. (2023). Age of onset and cumulative risk of mental disorders: A cross-national analysis of population surveys from 29 countries. *Lancet Psychiatry* 10 668–681. 10.1016/S2215-0366(23)00193-1 37531964 PMC10529120

[B60] Milad M. Igoe S. Lebron-Milad K. Novales J. (2009). Estrous cycle phase and gonadal hormones influence conditioned fear extinction. *Neuroscience* 164 887–895. 10.1016/j.neuroscience.2009.09.011 19761818 PMC2783784

[B61] Mineka S. Cook M. (1986). Immunization against the observational conditioning of snake fear in rhesus monkeys. *J. Abnorm. Psychol.* 95 307–318. 10.1037//0021-843x.95.4.307 3805492

[B62] Mineka S. Zinbarg R. (2006). A contemporary learning theory perspective on the etiology of anxiety disorders: It’s not what you thought it was. *Am. Psychol.* 61 10–26. 10.1037/0003-066X.61.1.10 16435973

[B63] Mitchell J. Vincelette L. Tuberman S. Sheppard V. Bergeron E. Calitri R. (2024). Behavioral and neural correlates of diverse conditioned fear responses in male and female rats. *Neurobiol. Stress* 33:100675. 10.1016/j.ynstr.2024.100675 39391589 PMC11465128

[B64] Nath T. Mathis A. Chen A. Patel A. Bethge M. Mathis M. (2019). Using DeepLabCut for 3D markerless pose estimation across species and behaviors. *Nat. Protoc.* 14 2152–2176. 10.1038/s41596-019-0176-0 31227823

[B65] Nelson A. Thur K. Cassaday H. (2012). Dopamine D1 receptor involvement in latent inhibition and overshadowing. *Int. J. Neuropsychopharmacol.* 15 1513–1523. 10.1017/S1461145711001751 22176724 PMC3496172

[B66] Orsini C. Willis M. Gilbert R. Bizon J. Setlow B. (2016). Sex differences in a rat model of risky decision making. *Behav. Neurosci.* 130 50–61. 10.1037/bne0000111 26653713 PMC4738105

[B67] Park C. Ganella D. Kim J. (2017). Juvenile female rats, but not male rats, show renewal, reinstatement, and spontaneous recovery following extinction of conditioned fear. *Learn. Mem.* 24 630–636. 10.1101/lm.045831.117 29142058 PMC5688961

[B68] Park C. Ganella D. Perry C. Kim J. (2020). Dissociated roles of dorsal and ventral hippocampus in recall and extinction of conditioned fear in male and female juvenile rats. *Exp. Neurol.* 329:113306. 10.1016/j.expneurol.2020.113306 32283056

[B69] Pattwell S. Duhoux S. Hartley C. Johnson D. Jing D. Elliott M. (2012). Altered fear learning across development in both mouse and human. *Proc. Natl. Acad. Sci. U.S.A.* 109 16318–16323. 10.1073/pnas.1206834109 22988092 PMC3479553

[B70] Pattwell S. Lee F. Casey B. (2013). Fear learning and memory across adolescent development: Hormones and behavior special issue: Puberty and adolescence. *Horm. Behav.* 64 380–389. 10.1016/j.yhbeh.2013.01.016 23998679 PMC3761221

[B71] Perry C. Campbell E. Drummond K. Lum J. Kim J. (2021). Sex differences in the neurochemistry of frontal cortex: Impact of early life stress. *J. Neurochem.* 157 963–981. 10.1111/jnc.15208 33025572

[B72] Perry C. Ganella D. Nguyen L. Du X. Drummond K. Whittle S. (2020). Assessment of conditioned fear extinction in male and female adolescent rats. *Psychoneuroendocrinology* 116:104670. 10.1016/j.psyneuen.2020.104670 32334346

[B73] Polanczyk G. Salum G. Sugaya L. Caye A. Rohde L. (2015). Annual research review: A meta-analysis of the worldwide prevalence of mental disorders in children and adolescents. *J. Child. Psychol. Psychiatry* 56 345–365. 10.1111/jcpp.12381 25649325

[B74] Robinson-Drummer P. Stanton M. (2015). Using the context preexposure facilitation effect to study long-term context memory in preweanling, juvenile, adolescent, and adult rats. *Physiol. Behav.* 148 22–28. 10.1016/j.physbeh.2014.12.033 25542890 PMC4782599

[B75] Rudy J. (1993). Contextual conditioning and auditory cue conditioning dissociate during development. *Behav. Neurosci.* 107 887–891. 10.1037//0735-7044.107.5.887 8280399

[B76] Rudy J. Sutherland R. (1995). Configural association theory and the hippocampal formation: An appraisal and reconfiguration. *Hippocampus* 5 375–389. 10.1002/hipo.450050502 8773252

[B77] Shalev U. Feldon J. Weiner I. (1998). Gender- and age-dependent differences in latent inhibition following pre-weaning non-handling: Implications for a neurodevelopmental animal model of schizophrenia. *Int. J. Dev. Neurosci.* 16 279–288. 10.1016/s0736-5748(98)00031-8 9785124

[B78] Stock H. Caldarone B. Abrahamsen G. Mongeluzi D. Wilson M. Rosellini R. (2001). Sex differences in relation to conditioned fear-induced enhancement of morphine analgesia. *Physiol. Behav.* 72 439–447. 10.1016/s0031-9384(00)00426-1 11274689

[B79] Velasco E. Florido A. Milad M. Andero R. (2019). Sex differences in fear extinction. *Neurosci. Biobehav. Rev.* 103 81–88. 10.1016/j.neubiorev.2019.05.020 31129235 PMC6692252

[B80] Westbrook R. F. Good A. J. Kiernan M. J. (1994). Effects of the interval between exposure to a novel environment and the occurrence of shock on the freezing responses of rats. *Q. J. Exp. Psychol. B* 47 427–446. 10.1080/1464074940840136 7809406

[B81] Westwood F. (2008). The female rat reproductive cycle: A practical histological guide to staging. *Toxicol. Pathol.* 36 375–384. 10.1177/0192623308315665 18441260

[B82] Wiltgen B. Sanders M. Behne N. Fanselow M. (2001). Sex differences, context preexposure, and the immediate shock deficit in Pavlovian context conditioning with mice. *Behav. Neurosci.* 115 26–32. 10.1037/0735-7044.115.1.26 11256449

[B83] Zbukvic I. Park C. Ganella D. Lawrence A. Kim J. (2017). Prefrontal dopaminergic mechanisms of extinction in adolescence compared to adulthood in rats. *Front. Behav. Neurosci.* 11:32. 10.3389/fnbeh.2017.00032 28275342 PMC5319962

[B84] Zeidan M. Igoe S. Linnman C. Vitalo A. Levine J. Klibanski A. (2011). Estradiol modulates medial prefrontal cortex and amygdala activity during fear extinction in women and female rats. *Biol. Psychiatry* 70 920–927. 10.1016/j.biopsych.2011.05.016 21762880 PMC3197763

[B85] Zuckerman L. Rimmerman N. Weiner I. (2003). Latent inhibition in 35-day-old rats is not an “adult” latent inhibition: Implications for neurodevelopmental models of schizophrenia. *Psychopharmacology* 169 298–307. 10.1007/s00213-003-1460-8 12827344

